# BdSL47: A complete depth-based Bangla sign alphabet and digit dataset

**DOI:** 10.1016/j.dib.2023.109799

**Published:** 2023-11-11

**Authors:** S M Rayeed, Sidratul Tamzida Tuba, Hasan Mahmud, Mumtahin Habib Ullah Mazumder, Saddam Hossain Mukta, Kamrul Hasan

**Affiliations:** a Systems and Software Lab (SSL), Department of Computer Science and Engineering (CSE), Islamic University of Technology (IUT), Board Bazar, Gazipur 1704, Bangladesh; b Department of Computer Science and Engineering (CSE), United International University (UIU), United City, Madani Avenue, Dhaka1212, Bangladesh

**Keywords:** Sign language recognition, Bangla sign language, Bangla sign alphabet dataset, Depth information, Hand key-points, Hand landmark model, MediaPipe

## Abstract

Sign Language Recognition (SLR) is crucial for enabling communication between the deaf-mute and hearing communities. Nevertheless, the development of a comprehensive sign language dataset is a challenging task due to the complexity and variations in hand gestures. This challenge is particularly evident in the case of Bangla Sign Language (BdSL), where the limited availability of depth datasets impedes accurate recognition. To address this issue, we propose BdSL47, an open-access depth dataset for 47 one-handed static signs (10 digits, from ০ to ৯; and 37 letters, from অ to ँ) of BdSL. The dataset was created using the MediaPipe framework for extracting depth information. To classify the signs, we developed an Artificial Neural Network (ANN) model with a 63-node input layer, a 47-node output layer, and 4 hidden layers that included dropout in the last two hidden layers, an Adam optimizer, and a ReLU activation function. Based on the selected hyperparameters, the proposed ANN model effectively learns the spatial relationships and patterns from the depth-based gestural input features and gives an F1 score of 97.84 %, indicating the effectiveness of the approach compared to the baselines provided. The availability of BdSL47 as a comprehensive dataset can have an impact on improving the accuracy of SLR for BdSL using more advanced deep-learning models.

Specifications Table

**Table:** 

Subject	Computer Vision and Pattern Recognition, Human-Computer Interaction, Sign Language Recognition
Specific subject area	Construction of Bangla Sign language Dataset that contains depth information extracted via MediaPipe framework (an open-source library for computer vision tasks) from Bangla alphabet sign and digit sign images. The depth information of the hand sign images can be utilized by different machine learning models to improve the sign language recognition accuracy for effective Human-Computer Interaction applications.
Data format	Raw, Analyzed, Filtered
Type of data	Image, CSV files
Data collection	The dataset samples were collected using a general-purpose webcam (60 fps, 640 × 480 resolution) from 10 signers with varying ages (14 years-62 years), gender (5 males and 5 females), hand shapes, skin colors, and illumination conditions. We also considered other variations like hand rotation, scaling, translation of hand signs, hand rotation, and orientation with different lighting ambiance and backgrounds. Out of 150 samples for each sign, we have finalized 100 samples based on the correct detection of hand key-points provided by the MediaPipe library (Version v0.10.5). We have stored the normalized values of the 21 hand key-points that use min-max normalization ranging values from 0 to 1 in the CSV files for each sign. So total we have 47000 input Read Green Blue (RGB) images, 470 CSV files containing 47 signs of Bangla sign and the Bangla sign alphabet (37 (অ to ँ)) digits (10 (০ to ৯)).
Data source location	Systems of Software Lab (SSL), Department of Computer Science and Engineering (CSE), Islamic University of Technology (IUT), OIC, Dhaka, Bangladesh. Department of Computer Science and Engineering (CSE), United International University (UIU), Dhaka, Bangladesh.
Data accessibility	Repository name: Mendeley Data Data identification number: 10.17632/pbb3w3f92y.3 Direct URL to data: https://data.mendeley.com/datasets/pbb3w3f92y/3
Related research article	

## 1 Value of the Data

• BdSL47 is a comprehensive dataset that can be a valuable resource for the researchers working on computer vision-based Bangla sign language recognition. The researchers and developers can explore the use of multimodal deep learning architectures to correctly identify Bangla hand signs because the dataset contains both RGB images and depth key-points of each sign for analysis.
• The dataset contains Bangla hand signs of digits and alphabets in different challenging conditions that reflect real-life scenarios and impose challenges for the researchers and developers. Moreover, this is the first-ever complete and large open-source dataset on Bangla sign alphabets and sign digits. The dataset can be used for benchmarking and comparing the performances of different machine learning models including traditional to advanced deep learning-based models.
• The dataset can be used by researchers and academicians to practice machine learning and pattern recognition tools and techniques, specifically for depth-based Bangla sign language recognition. Using the dataset AI-based Bangla sign language tools or applications can be developed by the software developers. The deaf and mute community can benefit from communicating through sign language translation generated automatically by understanding the constituting signs.
• As the dataset is made publicly available along with the test code to run, the other researchers will get a reference of this dataset for new algorithm development, performing comparative studies, doing feature engineering, and so on.

## 2 Data Description

The BdSL47 dataset consists of one-handed static hand gestures of 47 signs, with 37 of the sign alphabet (from sign অ to sign ँ), and 10 of sign digits (from sign ০ to sign ৯). The dataset includes a total of 47,000 jpg images, and 470 CSV files containing normalized 3D coordinate values of 21 predefined hand key points. Hand key points were extracted from RGB images using the MediaPipe framework (Version v0.10.5). The dataset was constructed by collecting Bangla alphabet signs from 10 users in a controlled setting. The description of the dataset is given in Table 1.

**Table 1:** Dataset Statistics of the proposed BdSL47 Dataset.

No. of input images	47000	Image resolution	640 × 480
No. of users	10 (5 male and 5 female)	Sign type	Static, 1-handed
No. of total signs	47	No. of csv files	470
No. of alphabet signs	37 (অ to ँ)	No. of samples per csv	100
No. of digit signs	10 (০ to ৯)	No. of input features	63
Image type	RGB	No. of output labels	47

We employed varying factors of the users like age, gender, hand shape, and skin color. We have also incorporated different challenges while collecting data like scaling, translation, hand rotation, hand orientation, lighting ambiance, and background. Table 2 shows the image samples of 47 signs of the proposed BdSL47 dataset:

**Table 2:** Proposed BdSL47 dataset (Sign Alphabet: 37 Signs [from Sign 00 (অ) to 36 (ँ)] and Sign Digits: 10 Signs [from Sign 37 (০) to 46 (৯)]).

Using the MediaPipe framework, we stored the normalized *x, y*, and *z* values for each of the 21 hand key-points detected from a sample. For normalizing the values of x and y coordinates, MediaPipe uses min-max normalization where all values are scaled in the range from 0 to 1. For storing depth values, standardization has been used with a mean of 0, and a standard deviation of 1, where the smaller the value, the closer the landmark is to the camera. For 100 samples of a sign from a user, we created one CSV file, consisting of the normalized 3D (*x, y, z*) coordinate values of 21 hand key-points (0 to 20), having 65 columns (including name and label). A sample CSV file is given below in Table 3:

**Table 3:** Sample CSV file (First Column: Sample name; Columns x00, y00, z00, …, x20, y20, z20: 3D values of 21 hand key-points; Last Column: Label).

Name	x_00_	y_00_	z_00_	…	…	x_20_	y_20_	z_20_	Label
1.jpg	0.5268	0.7077	−0.0015	…	…	0.6331	0.3030	−0.0290	5
2.jpg	0.5248	0.6950	−0.0017	…	…	0.6325	0.2845	−0.0215	5
3.jpg	0.5299	0.7029	−0.0017	…	…	0.6290	0.2685	−0.0353	5
…	…	…	…	…	…	…	…	…	5
…	…	…	…	…	…	…	…	…	5
98.jpg	0.4833	0.7143	−0.0037	…	…	0.3734	0.3343	−0.0637	5
99.jpg	0.4827	0.7285	−0.0064	…	…	0.3590	0.3404	−0.0297	5
100.jpg	0.4894	0.7471	−0.0060	…	…	0.3514	0.3380	−0.0384	5

From Table 3, we can see that the x, y, z coordinate values are stored in separate 63 columns, these columns are the skeleton of our tabular dataset. Since a CSV file contains information about 100 images of a sign collected from one user, there are 100 rows (without heading).

The proposed BdSL47 dataset is publicly available in the Mendeley Data repository, https://data.mendeley.com/datasets/pbb3w3f92y/3. Going to the link, there are two folders named as “Bangla Sign Language Dataset - Sign Alphabets” and “Bangla Sign Language Dataset - Sign Digits”. Under each folder, user-wise folders are given that contain sign images (input images (raw images in jpg format) and CSV files (normalized 3D coordinates of 21 hand keypoints with corresponding class label). All the files of around 1.02GB are available for direct download using the ‘Download All’ option by going to the DOI: 10.17632/pbb3w3f92y.3. The images and CSV files can be easily read or processed using Python or any other programming language (Python 3.10.3). Please cite our dataset if you have used it in your research following the format, “Rayeed, S M; Tuba, Sidratul Tamzida; Mahmud, Hasan; Mazumder, Mumtahin Habib Ullah; Hossain, Md. Saddam; Hasan, Md. Kamrul (2023), “BdSL47: A Complete Depth-based Bangla Sign Alphabet and Digit Dataset”, Mendeley Data, V3, doi: 10.17632/pbb3w3f92y.3”. The files associated with this dataset are licensed under a Creative Commons Attribution 4.0 International license. The link to the code can be found here: https://github.com/SMRayeed/BdSL47-Recognition

## 3 Experimental Design, Materials and Methods

This section describes the process of generating the BdSL47 dataset and the benchmarking results. In our previous work [1], we followed a similar approach of constructing a Bangla sign digit dataset consisting of 10 Bangla digits. In this work, we have merged the sign digits with the sign alphabet (37 letters) to make a complete BdSL dataset. The total number of labels turns to 47. This process goes through several phases, which broadly can be categorized into image sample collection and preprocessing phase and classification. A detailed approach involves the following steps.

### 3.1 Image sample collection

The first step of constructing the BdSL alphabet dataset was to collect RGB image samples. We employed 10 signers of varying ages and gender for this purpose. Prior to taking samples from the signers, we took their informed consent in written form. From a signer, more than 150 samples were captured for every sign (from sign অ to sign ँ), from which 100 that could most accurately detect the hand key-points were finalized. Taking into account that some frames may yield faulty or no hand detection due to darker lighting ambiance, blurry hand signs, lack of focus, and hand movement while capturing, some extra samples were taken. The samples were collected via a general-purpose webcam (60 fps, 640 × 480 resolution). Our primary focus was to capture the user's hand, hence, while taking samples, only the user's hand was in the field of view of the camera, and therefore no explicit hand segmentation was used.

**Fig. 1:**
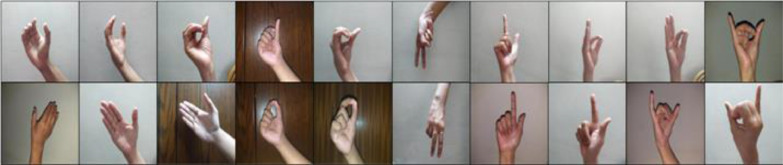
Variations in user and environment setup during sample collection.

***Variation in sample collection:*** To make the sign dataset challenging, the image samples were collected considering the user variations and environment variations which include different factors like the expertise level of the signers, their age, gender, hand shapes, and skin colors. Signers were different from each other in at least one of the factors. We anonymously collected the samples, from signers with an age range from 14 to 62, among whom 5 were male and 5 were female. We also considered environmental variations in different aspects like scaling, translation, hand rotation, hand orientation, lighting ambiance, and background. Examples of such variations are shown in Fig. 1.

After the image sample collection, we have gone through the following preprocessing steps:

• Process the input image through MediaPipe to detect correct hand key-points and generate a corresponding output image consisting of overlaid hand key-points.
• Removing samples with faulty key-points by manually checking the samples through Bangla Sign professional
• Resizing the gestural image into 640 × 480, min-max normalization, and standardization of the depth values to generate the CSV files
• Merging the Bangla Sign Alphabet dataset with the Sign Digit Dataset and correcting the output labels to construct a complete BdSL47

These steps are elaborated in the subsequent sections.

### 3.2 Hand key-points detection

**Fig. 2:**
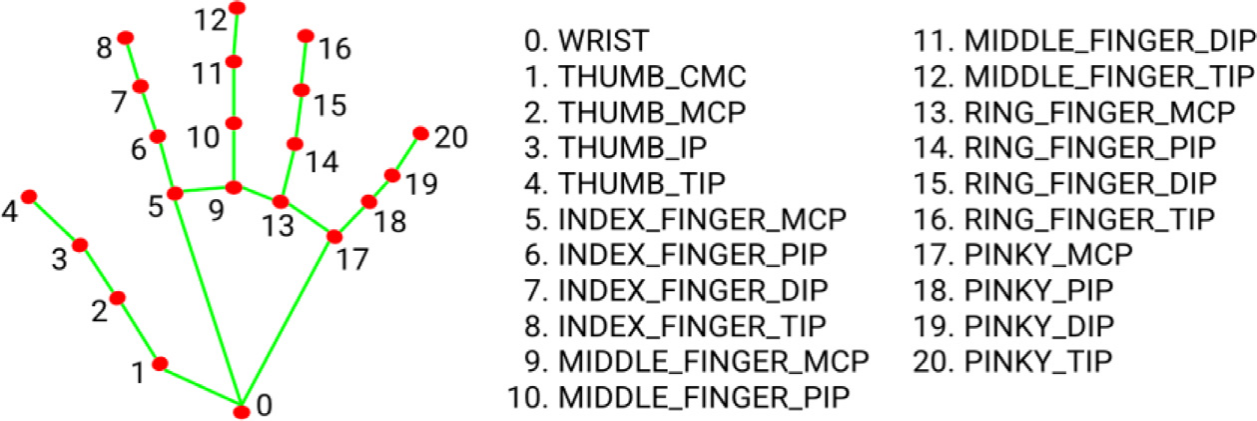
Predefined 21 Hand Key-points of MediaPipe Hand Module.

**Fig. 3:**
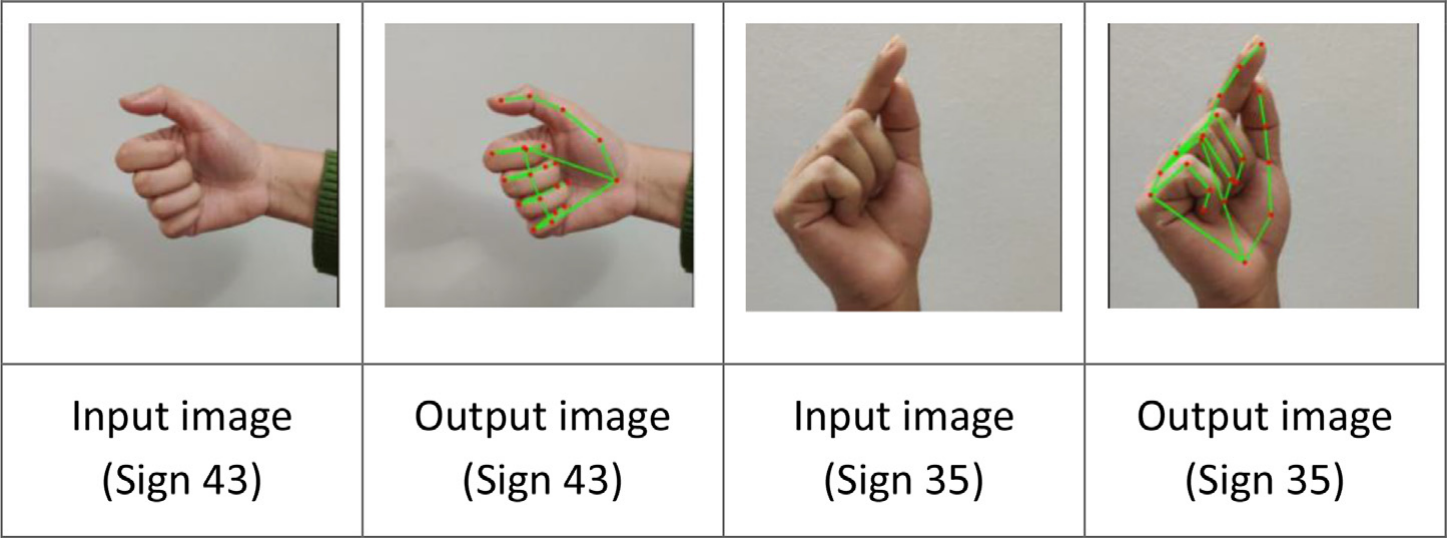
MediaPipe generated sample images with respective hand key-points.

After capturing the RGB images, the image frames were processed via the MediaPipe framework for hand-tracking and hand key-points detection. MediaPipe Hands (Fig. 2), runs two underlying models – the palm detection model, BlazePalm, for hand detection, and Hand Landmark Model for 21 hand key-points detection. The image samples were processed through MediaPipe individually. Fig. 3 depicts how we have used the MediaPipe framework for detecting hand key-points and generating a corresponding output image for each sample.

### 3.3 Removal of samples with faulty key-points detection

**Fig. 4:**
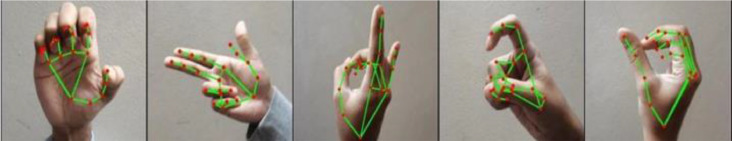
Discarded output samples with faulty hand key-points.

One of the limitations we have faced while working with MediaPipe is, in the case of a few samples, processing via MediaPipe sometimes resulted in faulty detections of hand key-points, mostly due to darker lighting ambiance, user-hand being blurred, and complex background. Because of this, after generating the output images, we had to manually check the samples for such faulty cases to discard them. After scrutinization, 100 samples that accurately detected hand key-points were selected for each sign from every signer. Some examples are shown in Fig. 4.

After that, finalized samples were validated and verified by a Bangla sign language professional.

### 3.4 Data files generation

After manual checking and selection, the image samples were resized into a standard size, 640 × 480 in case of landscape images and 480 × 640 in case of portrait images. After final processing of these images via MediaPipe that includes normalization (min-max), and standardization of the depth values. After all these steps we generate the CSV files consisting of 3D coordinate points, names, and labels as shown in Table 2.

### 3.5 Constructing BdSL47: merging of sign alphabet dataset with sign digit dataset

As mentioned earlier, in our previous work [1] on Bangla sign digits, we constructed a dataset of 10 signs of Bangla sign digits. After completion of the sign alphabet dataset, to construct a single complete dataset, we merged the two datasets, which now consist of 47 signs (10 digits and 37 letters) and have been named as ‘BdSL47’. However, while merging two datasets, we had to make some basic adjustments in labeling. In the alphabet dataset, we have labeled the samples from 0 to 36 for 37 signs of the Bangla sign alphabet, and in the BdSL digit dataset, the images were also labeled from 0 to 9 for the 10-digit signs; therefore, we had to relabel the samples from sign digits for classification purpose in the merged single dataset to avoid mislabelling which would significantly decrease the accuracy. The 10 sign digits (from sign ০ to ৯) have been relabelled from 37 to 46, following Table 1. Therefore, in the single complete dataset, samples for sign ‘অ’ to sign ’ ँ’ has been labeled from 0 to 36 and samples for sign ‘০’ to ‘৯’ has labeled from 37 to 46 making the total 47 labels for the dataset.

**Fig. 5:**
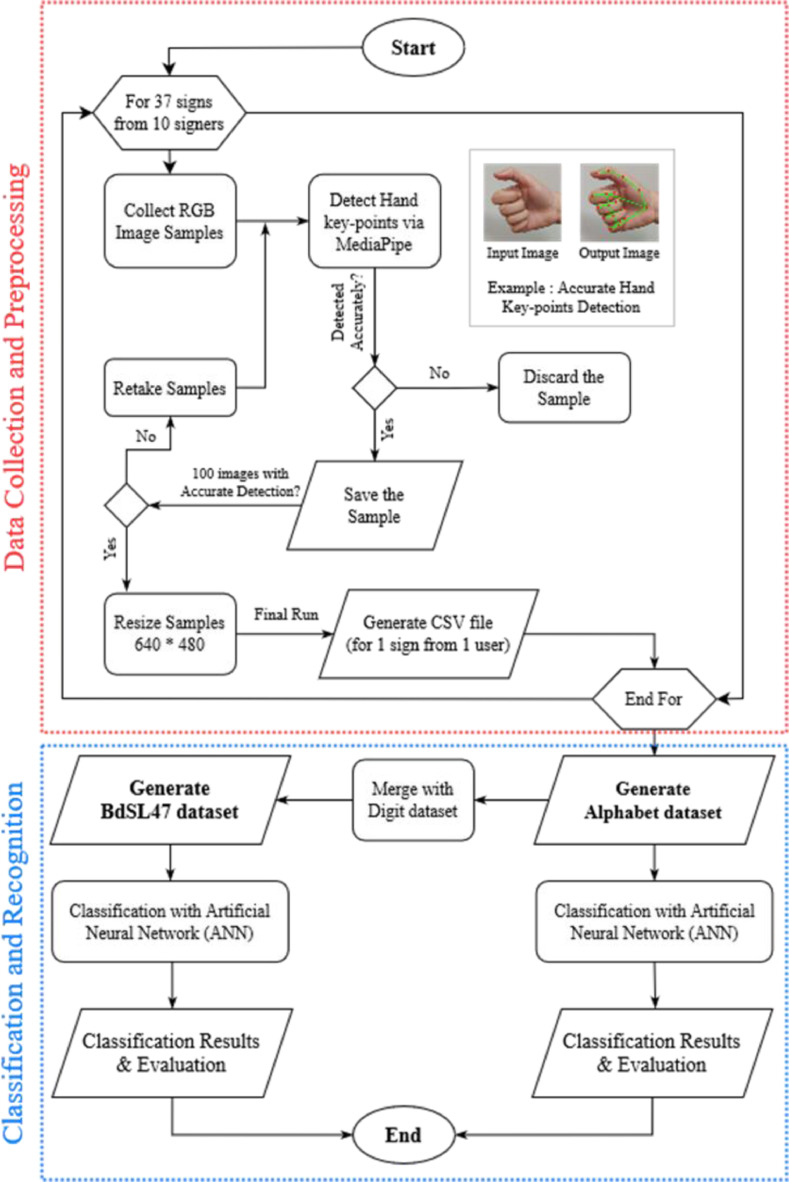
Flow diagram of BdSL47 dataset generation and classification steps .

The overall process of the proposed approach is given in the following Flow Diagram in Fig. 5.

### 3.6 Classification and analysis

To give a baseline of our proposed dataset, we applied both traditional and deep learning-based machine learning algorithms and analyzed the classification results. The detailed process has been described in the subsequent sections:

#### 3.6.1 Classification using traditional models

Here we discuss the results we achieved out of the classical machine learning models. On our proposed BdSL47 dataset, we ran four traditional machine learning models, KNN, SVM, LR, and RFC, as well as two ensembling methods, Hist-GBC and Light-GBM.

As we have shown in Table 3, our proposed BdSL47 dataset is constructed from 47000 RGB images containing 47 static one-handed signs, collected from 10 signers. After data pre-processing using via MediaPipe library, 470 corresponding CSV files were generated. On the other hand, the sign alphabet dataset is constructed from 37000 images, therefore 370 corresponding CSV files were generated after processing.

These CSV files contain *x, y*, and depth coordinate values of 21 hand key-points extracted from these image samples and constitute 21 × 3, or 63 input features. It is a multilabel classification problem, with 47 output labels for the BdSL47 dataset and 37 labels for the sign alphabet dataset. Prior to classification, a 10-fold cross-validation set was prepared, and the dataset was split into a standard train-test ratio of 80:20. Table 4 and Table 5 respectively show the results for KNN, SVM, LR, RFC, Hist-GBC, and Light-GBM on the BdSL47 dataset and the sign alphabet dataset:

**Table 4:** Classification results for traditional and ensemble machine learning classifiers on the BdSL47 dataset (47 Labels).

Classifier	Accuracy (%)	Precision (%)	Recall (%)	F1 Score (%)	Time Taken (Seconds)
KNN	96.40	96.43	96.40	96.41	6.01
SVM	93.65	93.76	93.65	93.65	49.65
LR	82.60	82.75	82.60	82.57	63.54
RFC	96.84	96.84	96.84	96.85	19.97
HistGBC	96.49	96.52	96.49	96.49	168.89
Light-GBM	96.99	97.01	96.99	96.99	79.52

**Table 5:** Classification results for traditional and ensemble machine learning classifiers on the sign alphabet dataset (37 Labels).

Classifier	Accuracy (%)	Precision (%)	Recall (%)	F1 Score (%)	Time Taken (Seconds)
KNN	98.49	98.50	98.49	98.49	3.52
SVM	96.55	96.58	96.55	96.55	26.43
LR	86.31	86.45	86.31	86.30	59.38
RFC	98.53	98.53	98.53	98.54	12.11
HistGBC	98.81	98.82	98.81	98.81	123.53
Light-GBM	98.84	98.85	98.84	98.84	54.68

In the KNN model, we set the value of “n_neighbors” to 3, so that for each test data point, the KNN algorithm will consider the 3 nearest data points in the training set to predict its label. This is the optimal value of "n_neighbors" which we obtained using the cross-validation approach. In the SVM model, we used the RBF kernel (Radial Basis Function) as our dataset is linearly inseparable. This kernel projects the input data into a high-dimensional feature space where it becomes linearly separable and helps to find the optimal hyperplane for SVM. Lastly, in the RFC model, the value of “n_estimators” (i.e., the number of decision trees in the forest) is set to 47, which is the optimal value obtained from the empirical research study.

We also used the gradient boosting ensemble technique, where multiple weak models are combined to achieve better performance. In Hist-GBC, histogram-based decision trees are used as the weak models, that are constructed using gradient-based histogram binning. This GBC can handle sparse data and is highly memory efficient, making it particularly useful when working with huge datasets with many features (like in our case). We also used Light-GBM, which is designed to be scalable for large-scale classification tasks. The model uses gradient-based one-side sampling for selecting only the instances with large gradients to update the model and exclusive feature bundling for grouping similar features in order to reduce the number of less-significant features.

***Result analysis:*** The traditional classifiers showed excellent performance on our proposed datasets, within significantly lower computational time. The incorporation of depth information with the x and y coordinate values ensures a significant improvement in distinguishability which is reflected in the results. Among these 3 classifiers, SVM takes more time, since the dimension of the feature space is very high to get linearly separable data (due to the high number of input features).

Overall, the classification results are satisfactory, considering the computational time and accuracy scores. It goes with our motivation of constructing a dataset that can be classified using baseline models in a lower computational time. Later, we designed a simple ANN model in order to improve the accuracy further, which is discussed in the next section.

#### 3.6.2 Classification using proposed ANN model

We designed an ANN model that consists of 63 nodes in the input layer (for 213, or 63 input features), 4 hidden layers (with dropouts in the last two), and an output layer. The number of output layer nodes varies based on the number of classes in the datasets, that's why there are 47 and 37 nodes in the output layers of BdSL47 and sign alphabet datasets, respectively.

As a proposed model, we tried to compare its results with traditional machine learning models. A similar approach of comparing novel architectures with traditional classifiers has been followed in [2], [3], [4], [5]

The number of neurons in input layers was determined based on an empirical study. In order to prevent overfitting, we applied dropout regularization to the last 2 hidden layers, with a rate of 0.33 and 0.25 respectively. In the hidden layers, we used ‘Leaky ReLU’ as the activation function (Leaky ReLU is a variation of the ReLU function that prevents the dying ReLU problem, by allowing small negative values to pass through instead of setting them to 0). For the compilation phase, we set the activation function to ‘SoftMax’ as this was a multi-label classification problem.

***Optimizer, learning rate, and loss function:*** The accurate choice of optimization algorithm has a significant impact on the classification results in machine learning and computer vision applications. Among the contemporary deep learning optimizers e.g., Adam, Stochastic Gradient Descent (SGD), AdaGrad, and RMSProp, the first one suits the best in our case. The Adam optimization algorithm is an upgraded extension of SGD which, unlike SGD, does not maintain a constant learning rate through training, but learns to adapt and updates the learning rate for each network weight individually. It can also be viewed as an extension of the RMSProp because it not only relies upon the first moment for adapting learning rates like RMSProp, but it also uses the second moment of gradients [13]. For its ubiquity, efficiency, and accuracy, it has been adapted as a benchmark for deep learning papers and recommended as a default optimization algorithm.

In our proposed method, we have used the Adam optimizer with a learning rate of 0.001. Learning rate is a key hyperparameter to set while training a neural network, because a low learning rate results in slow progress in training and tiny updates to network weights, whereas a high learning rate has a risk of hastily converging to a suboptimal solution instead of the optimal solution. So we chose the recommended learning rate of Adam optimizer, which performed well on our dataset.

**Fig. 6:**
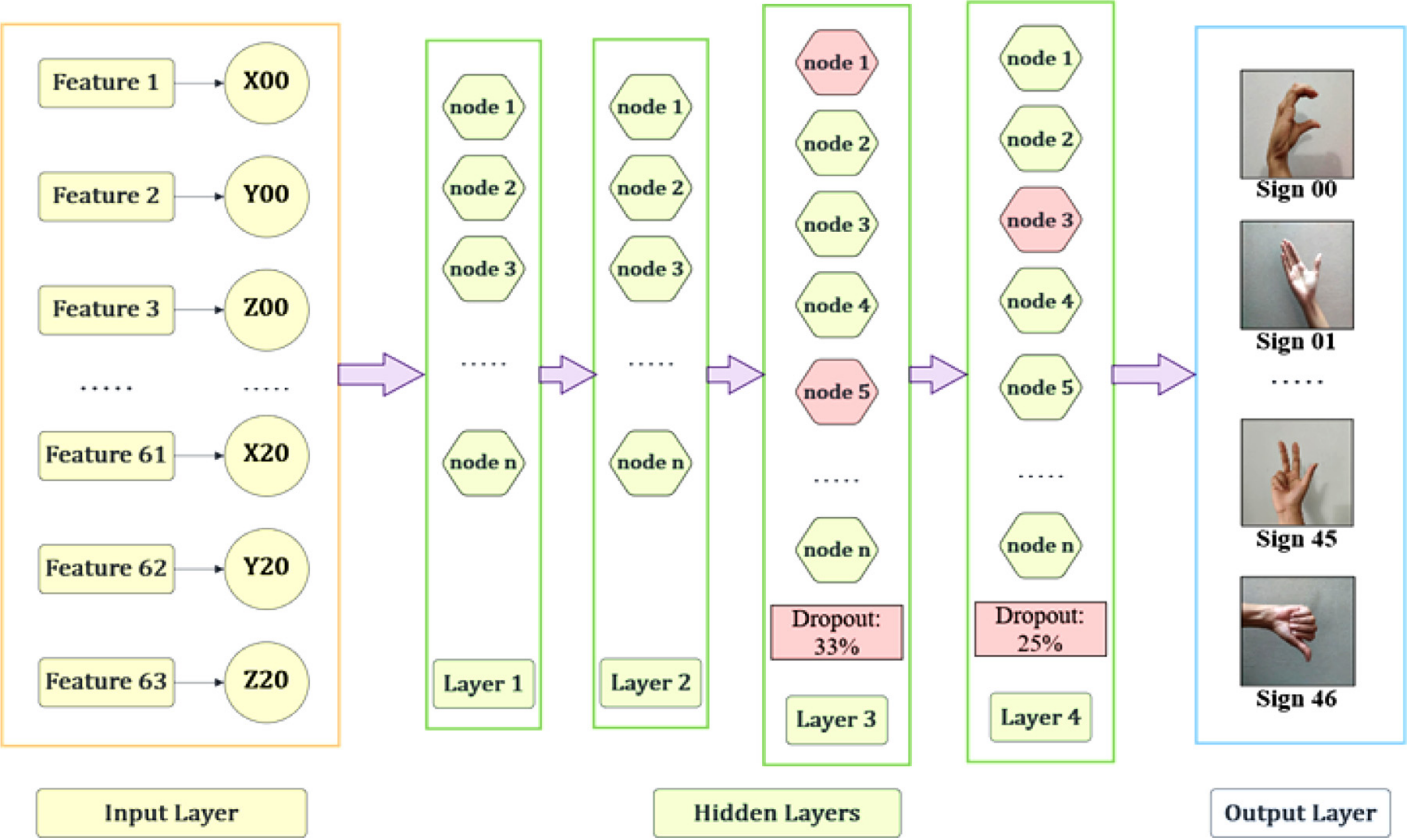
Architectural diagram of the proposed ANN model.

In terms of the loss function, for a multilabel classification using neural networks, most commonly used loss functions are categorical cross entropy (CCE) and sparse categorical cross entropy (SCCE). CCE is used for cases where soft probabilities exist that allow a sample to have multiple probabilities (e.g. 0.6 probability of belonging to label x and 0.4 to label y). On the contrary, SCCE is used when output labels are mutually exclusive. As our case matches with the latter one, we have used sparse categorical cross entropy (SCEE) as the loss function. Parameters and hyperparameters set in the ANN classification are listed in Table 6. An architectural diagram of the model is shown in Fig. 6.

**Table 6:** Configuration of parameters and hyperparameters in our ANN model.

Optimizer	Adam	Number of Dropout Layers	2
Learning Rate	**0.001**	Number of Hidden Layers	**4**
Loss Function	**SCEE**	Activation Function (Hidden Layers)	**Leaky ReLU**
Epochs	**100**	Activation Function (Compilation)	**Softmax**

**Fig. 7:**
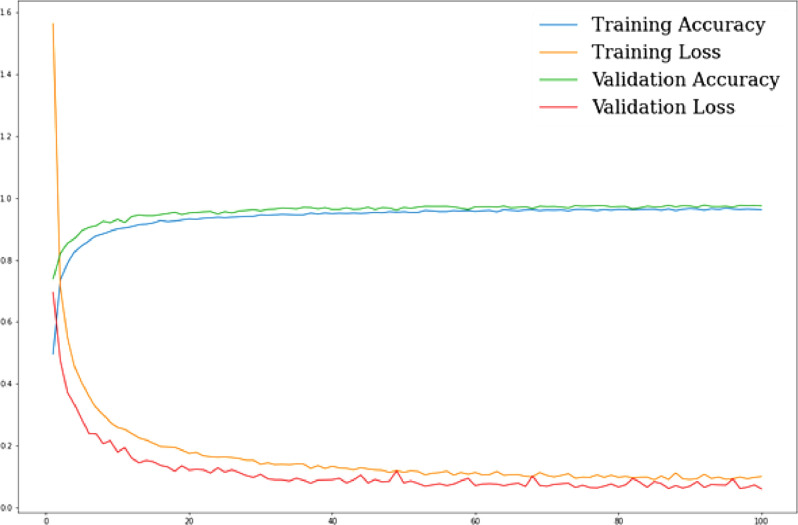
Performance of ANN on training and validation set: BdSL47 (47 labels).

**Fig. 8:**
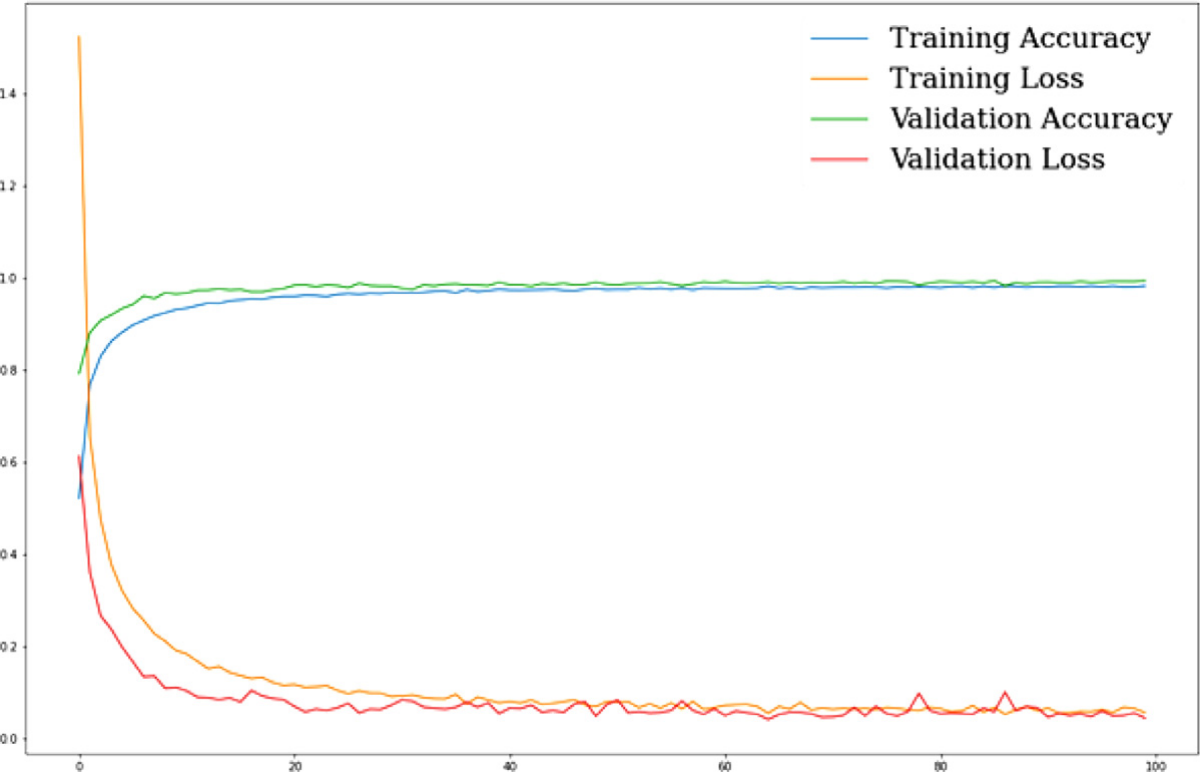
Performance of ANN on training and validation set: alphabet dataset (37 labels).

**Fig. 9:**
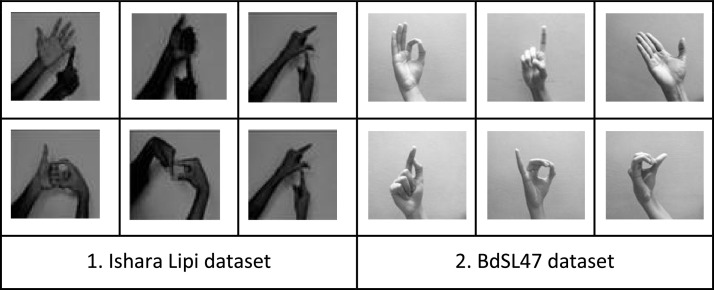
Comparison between grayscale images of 1. Ishara Lipi dataset and 2. BdSL47 dataset.

***ANN classification results****:* After designing the ANN model, first we ran it on the BdSL47 dataset, and then separately on the sign alphabet dataset, with the necessary changes in parameters. Like the previous cases, the train-test ratio was set to 80:20, and the ANN model ran for 100 epochs. After 100 epochs, the model gave 96.24 % training accuracy, 97.46 % validation accuracy, and 97.84 % testing accuracy. The total computational time taken for the entire process was about 262 seconds. After this, we repeated the same process on the sign alphabet dataset, which resulted in 98.30 % training accuracy, 99.46 % validation accuracy, and 99.41 % testing accuracy, in 192 seconds. Fig. 7, Fig. 8 show the performance of our proposed ANN model on training and validation data, respectively for BdSL47 and sign alphabet dataset:

***Result analysis:*** The ANN model performed very well on both datasets and yielded better classification accuracy compared to traditional classifiers. As stated earlier, the normalized 3D coordinate values of our datasets ensure enhanced discernibility. Our proposed ANN model was able to learn the spatial relationships and patterns from the depth-based gestural features very efficiently and quickly, which is why we can see a steep increase (See Fig. 8: from 52.09 % to 88.22 % within the first 5 epochs). In terms of validation loss, after about 85 epochs, the model starts getting saturated. We have plotted the training loss and validation loss against the number of epochs (Figs. 8 and 9), from which we can see the validation loss stops decreasing after about 85 epochs.

***Individual and cumulative classification results and analysis:*** After classifying our datasets using the ANN model, we calculated classification results for the signs individually, and then cumulatively. Tables 7 and 8 show individual scores for the BdSL47 dataset and sign alphabet dataset respectively.

**Table 7:** Label-wise classification results of ANN model: BdSL47 dataset.

**Table 8:** Label-wise classification results of ANN model: alphabet dataset.

For calculating scores for the signs individually, we used the confusion matrix and extracted the values of True Positive (TP), True Negative (TN), False Positive (FP) and False Negative (FN) cases for each sign. Then we evaluated the performance metrics (Accuracy, Precision, Recall, F1-Score/F-measure) using the following equations:

**Table:** 

From these individual scores, we can see that the ANN model has made few misclassifications in cases of some particular signs (e.g. Sign 21, 26, 32, 38, 43, 44, 45), because some of the digit signs are almost identical to some of the letter signs. For example, sign 43 (৬) is very similar to sign 26 (দ). Because of this similarity, the coordinate values of these signs are quite close; hence, distinguishing output labels from input features becomes somewhat difficult. Table 9 below shows the proximity and the number of misclassifications of the signs.

**Table 9:** Identical signs in sign-digits and sign-alphabet dataset.

We also calculated the cumulative classification results for the datasets, for which we used two approaches, micro scores (i.e. micro accuracy, micro recall) and macro scores (i.e. macro precision, macro f1-score). Macro score is the simple arithmetic mean of individual scores. In contrast to that, micro score is a summary statistic that takes into account the performance of each individual class separately, giving equal weight to each class's score.

**Table:** 

Using the equations, we evaluated the cumulative results (Table 10).

**Table 10:** Cumulative Classification Results for the datasets using ANN model (A: Accuracy, P: Precision, R: Recall, F1: F1-Score).

Dataset	Micro-Scores				Macro-Scores			
	A	P	R	F1	A	P	R	F1
1. BdSL47	0.999	0.978	0.978	0.978	0.999	0.978	0.979	0.978
2. Alphabet	0.999	0.994	0.994	0.994	0.999	0.994	0.994	0.994

The ANN model resulted in excellent scores also in the case of cumulative data. From Table 10, we find that the micro scores are remarkably high, which means the model showed outstanding performance for individual classification. From the high macro scores, we can reliably address the point that the overall performance graph is also very consistent across both datasets. Moreover, we compared performance metrics’ scores between traditional classifiers and the proposed ANN model (Table 11).

**Table 11:** Comparison of micro & macro scores between ANN and traditional models: BdSL47 (A: Accuracy, P: Precision, R: Recall, F1: F1-Score).

Models	Micro-Scores				Macro-Scores			
	A	P	R	F1	A	P	R	F1
KNN	0.9985	0.964	0.964	0.964	0.9985	0.964	0.9642	0.964
SVM	0.9973	0.9365	0.9365	0.9365	0.9973	0.9366	0.9369	0.9362
RFC	0.9987	0.9684	0.9684	0.9684	0.9987	0.9683	0.9684	0.9683
LR	0.9926	0.8260	0.8260	0.8260	0.9926	0.8255	0.8261	0.8249
Hist-GBC	0.9985	0.9649	0.9649	0.9649	0.9985	0.9649	0.9651	0.9649
LGBM	0.9987	0.9699	0.9699	0.9699	0.9987	0.9700	0.9701	0.9700
**ANN**	**0.9991**	**0.9784**	**0.9784**	**0.9784**	**0.9991**	**0.9786**	**0.9792**	**0.9784**

### 3.7 Comparative analysis with other BdSL datasets

As mentioned earlier, several research works have been conducted on Bangla Sign Language, and authors have constructed a few datasets, mainly on sign alphabet. Table 12 shows a comparison among them:

**Table 12:** Comparison in dataset structure with previous BdSL datasets.

Ref	Type	No. of Signs	Sign Type	Dataset Size (Images)	Background and Lighting	Dataset Access
[6]	Digits Alphabet	46	Static 1-handed 2-handed	27600	Static	No
[7]	Digits Alphabet Others	52	Static 1-handed 2-handed	36400	Static	No
[8]	Alphabet	37	Static 1-handed	518	Static	No
[9]	Alphabet	10	Static 2-handed	100	Random	Yes
[12]	Alphabet	36	Static 2-handed	1200 (Augmented to 400K)	Random	Yes
[10]	Alphabet	36	Static 2-handed	1800	Static	Yes
[11]	Digits	10	Static 2-handed	1000	Static	Yes
[1]	Digits	10	Static 1-handed	10000 100 csv	Random	Yes
**Ours**	**Alphabet**	**37**	**Static** **1-handed**	**37000** **370 csv**	**Random**	**Yes**
**Ours**	**Digits** **Alphabet**	**47**	**Static** **1-handed**	**47000** **470 csv**	**Random**	**Yes**

***Comparative analysis****:* From Table 12, we can see that most of the BdSL datasets have been built based on 2-handed static alphabet signs, and they were all image datasets, on which mostly deep learning-based approaches were performed for classification. On the contrary, although we have an image dataset as a byproduct, our proposed BdSL47 is a tabular dataset based on normalized 3D (x, y, and z) coordinate values stored in 470 csv files. Because of the incompatibility, a completely equivalent comparison among the classification results is not possible. However, we tried to list the previous works on BdSL, with corresponding feature extraction techniques and classification models of the referenced datasets for a better overview in Table 13.

**Table 13:** Comparison in classification with previous BdSL datasets.

Ref	Type	No. of Signs	Feature Extraction Method	Classifier	Accuracy (%)
[6]	Digits Alphabet	46	FRB-RGB	Haar-KNN	95.67
[7]	Digits Alphabet Others	52	NOBV + WGV	NOBV + WGV	95.83
[8]	Alphabet	37	BSL-FTP	ANN	98.99
[9]	Alphabet	10	Faster R-CNN	CNN	98.20
[12]	Alphabet	36	CNN	VGG19	99.10
[10]	Alphabet	36	CNN	CNN	94.74
[11]	Digits	10	CNN	CNN	92.87
[1]	Digits	10	MediaPipe	SVM	98.65
**Ours**	**Alphabet**	**37**	**MediaPipe**	**ANN**	**99.91**
**Ours**	**Digits** **Alphabet**	**47**	**MediaPipe**	**ANN**	**99.97**

***Result analysis****:* From the comparison above, we can clearly see that our proposed dataset performed significantly better than others. Unlike most of these works, we used relatively simpler models for classification, which nevertheless provided very good results. First, traditional and ensemble models performed quite well and yielded decent scores in a very short computation time. Then we used our proposed ANN model, which shows remarkable scores in relatively less computational time.

Additionally, we have conducted experiments to analyze the effectiveness of depth information in both traditional and ANN models. For this, we trained the models without depth features, evaluated the scores, and compared the performance. In that case, there was a 3.4 % improvement in overall performance considering the depth features along with non-depth features.

We have also conducted a statistical analysis to evaluate the superiority of our proposed approach. Our machine learning models show an outstanding performance in predicting hand signs. To understand why these feature sets obtain the higher performance, it is necessary to demonstrate that our feature sets are distinguishable from each other and they are statistically significant. Towards this direction, we have applied a statistical test where we have 63 independent features (i.e., x and y are non-depth features and z is the depth feature) and one dependent variable. To determine the significance of the depth feature over the non-depth features, we have performed a Multivariate Analysis of Variance (MANOVA). MANOVA is a suitable method for testing differences in mean vectors across multiple groups or factors, and it allows us to test the overall significance of the model as well as the significance of individual variables. By using MANOVA, we have determined if there is a significant difference in the mean vectors of the non-depth features between groups based on the depth feature. After conducting the MANOVA test, we found statistical significance results of pr<0.0000. Thus, we accept the alternative hypothesis and reject the null hypothesis which indicates that these static Bangla signs have distinguishable feature sets. Therefore, our depth information is a good indicator to present these signs and distinguish them from one another. Though we have a high number of classes, our depth features separate them clearly.

### 3.8 Comparison with the reference dataset Ishara Lipi

As we considered Ishara Lipi [10] as our reference dataset, we delineated a comparative analysis between the two datasets. Ishara Lipi contains 2-handed static signs of the 36 letters, with 50 RGB image samples per sign. Prior to classification, the samples were cropped, resized to 128 × 128 pixels, and converted to Grayscale. Also, the images in Ishara Lipi were taken in front of a white background. For an exact comparative overview, we have also taken 50 samples per sign (with a white background), resized them, and converted them to Grayscale.

We followed the same CNN model as proposed in Ishara Lipi [10]. The 9-layer CNN architecture consists of two sets of convolution layers with max-pooling after each layer. In both convolution layers, the kernel size is 5 × 5, and ‘ReLU’ is used as the activation function. Here the loss function is categorical cross-entropy, and the optimizer is adam, with a learning rate of 0.001 %.

On our alphabet dataset, the CNN model gave better classification results with a lesser number of epochs. For a better overview, important factors in comparing the two datasets are given in Table 14.

**Table 14:** Accuracy Results for KNN, SVM, RFC on the BdSL47 dataset

Factors	Ishara Lipi dataset	Sign alphabet dataset
No. of Samples	36	37
Samples per sign	50	50
Total Samples	1800	1850
Sample Type	2-handed, Static	1-handed, Static
Background	White	White
Image Size	128 × 128	128 × 128
Image Type	Grayscale	Grayscale
Optimizer	Adam	Adam
Accuracy	94.74 %	98.92 %

***Result analysis:*** The classification results show that our dataset outperformed the Ishara Lipi dataset using the same CNN architecture, despite being homogeneous in structure and size. Because of the signs were two-handed in the Ishara Lipi dataset, these gestures were more complex for recognition.

Moreover, after resizing into 128 × 128 and converting to grayscale, due to lower lighting ambiance, some parts of several signs became too dark in the Ishara Lipi dataset. Compared to that, after resizing and converting to grayscale, the signs are more clearly visible and easily distinguishable in our dataset, which resulted in a significant increase in classification accuracy. Fig. 9 shows a comparison between grayscale images of the datasets.

## Limitations

The proposed dataset limits natural variations in the data due to controlled settings while collecting Bangla sign images. The results of the classification models show that both neural network models performed well on the proposed dataset, even with a high number of classes, as the hand key points were well-distinguished from one another. Moreover, the experiments we conducted through the proposed ANN model can be further extended by constructing new datasets using various augmentation techniques like adding noise, translating, scaling, rotating, etc. Then the performance of both ANN and deep learning-based architectures can be compared based on the augmented dataset.

In the future, we aim to make the dataset more challenging with dynamic Bangla signs and to explore other features for classification purposes, such as finger foldedness, the angle between fingers, etc. We can also investigate multimodal classification on our dataset. The dataset as of now includes only single-handed static signs, and future research may include two-handed signs, facial expression while producing signs, etc. This dataset has practical applications beyond theoretical research and could be used in the development of real-time Bangla sign recognition systems based on depth information.

## Ethics Statement

The research is entirely dependent on indistinguishable data collected from photographs of the palms of human subjects and there is no data collection based on invasive/clinical testing of human subjects. As the data were collected using a standard webcam there was no physical impact on the participants. Prior to data collection,

We have taken ethical approval (IREB reference number, IREB/2023/007) for this research by the Institutional Research Ethics Board (IREB) of United International University (UlU), Dhaka, Bangladesh.

## CRediT authorship contribution statement

**S M Rayeed:** Conceptualization, Methodology, Software, Validation, Investigation, Writing – original draft, Writing – review & editing. **Sidratul Tamzida Tuba:** Software, Resources, Data curation. **Hasan Mahmud:** Conceptualization, Methodology, Resources, Writing – original draft, Writing – review & editing, Validation, Investigation, Writing – original draft, Supervision, Project administration, Funding acquisition. **Mumtahin Habib Ullah Mazumder:** Data curation, Supervision, Project administration, Funding acquisition. **Saddam Hossain Mukta:** Project administration, Funding acquisition. **Kamrul Hasan:** Supervision, Project administration, Funding acquisition.

## Data Availability

BdSL47: A Complete Depth-based Bangla Sign Alphabet and Digit Dataset (Original data) (Mendeley Data)
